# Dual MNK/VEGFR2 Inhibitor JDB153 Enhances Immunotherapeutic Efficiency and Chemosensitivity in Lung Cancer

**DOI:** 10.1002/mco2.70155

**Published:** 2025-04-29

**Authors:** Maosen Xu, Li Xu, Tao Zhang, Xue Li, Ziqi Zhang, Ruolan Xia, Ning Jiang, Li Yang, Xiawei Wei

**Affiliations:** ^1^ Laboratory of Aging Research and Cancer Drug Target State Key Laboratory of Biotherapy National Clinical Research Center for Geriatrics West China Hospital Sichuan University Chengdu Sichuan China; ^2^ Jumbo Drug Bank Co., Ltd. Chengdu China

**Keywords:** eIF4F, inhibitor, JDB153, lung cancer, MNK, VEGFR2

## Abstract

Lung cancer continues to be the primary cause of cancer‐related mortality worldwide, with non‐small cell lung cancer (NSCLC) being the predominant type. Dysregulation of protein translation that participates in cell proliferation is an important factor to define oncogenic processes and cancer development. The eukaryotic initiation factor 4E (eIF4E) regulates ribosomal translation of proteins from mRNA, and the mitogen‐activated protein kinase interacting kinases (MNKs) is reported to be the only kinases that can phosphorylate eIF4E. Substantial previous work has proven that the MNK–eIF4E axis is usually dysregulated in many cancer types. Moreover, abnormal angiogenesis is essential for tumorigenesis and cancer progression, and vascular endothelial growth factors (VEGF) together with their receptors play multiple crucial roles in angiogenesis, especially VEGFR2. In this study, we report a novel dual MNK/VEGFR2 inhibitor named JDB153 and investigate its antitumor effects in NSCLC. JDB153 can effectively inhibit the phosphorylation of eIF4E and VEGFR2, suppress proliferation, migration and invasion, promote apoptosis, and induce cycle arrest of lung cancer cells. Importantly, JDB153 exhibits antitumor activity and synergizes with anti‐PD‐1 therapy and cisplatin with reliable safety. Our findings reveal the potential value of JDB153 in lung cancer as monotherapy or in combination with immunotherapy and chemotherapy, with the hope to provide a novel combinational strategy for NSCLC treatment clinically.

## Graphical abstract

JDB153 is a novel dual MNK/VEGFR2 inhibitor that exhibits effective therapeutic activity against NSCLC with favorable safety. It inhibits the phosphorylation of eIF4E and VEGFR2 dramatically and suppresses the growth and proliferation of both LL2 and H460 cells. JDB153 can promote cell apoptosis, induce cell cycle arrest, and inhibit tumor neovascularization. In the experiments in vivo, JDB153 in combination with PD‐1 Ab or cisplatin exhibits synergetic antitumor capacity with good tolerance, which would be complementary to current antitumor approaches and aid the optimization of NSCLC treatment

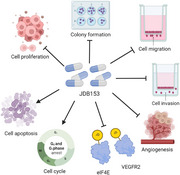

## 1 Introduction

Lung cancer is one of the most frequently occurring malignant tumors and the leading cause of cancer‐related mortality worldwide [1, 2]. According to cell origin, non‐small cell lung cancer (NSCLC) is the main type and approximately accounts for 85% among all lung cancer cases. Despite recent advancements in cancer treatment, NSCLC typically exhibits a poor prognosis with low long‐term survival rates [3, 4]. Currently, platinum‐based doublet chemotherapy remains the cornerstone of first line treatment for advanced NSCLC patients. However, unexpected drug resistance and significant adverse effects usually limits their further application, which makes combined therapy promising strategies [5, 6]. Therefore, it is necessary to explore effective therapeutic regimens for the treatment of NSCLC.

Within eukaryotic cells, protein synthesis emerges as a pivotal and intricately regulated process, exerting profound influence over diverse physiological phenomena including cellular proliferation, differentiation, and growth. Translation control is a tightly regulated process in protein synthesis, but the deregulation of protein synthesis usually participates in tumorigenesis and cancer development [7]. The translation process consists of four consecutive stages including initiation, elongation, termination as well as ribosome recycling, with translation initiation emerging as a focal point of investigation. Meanwhile, the translation control process of eukaryotic gene expression is mainly regulated at the initiation stage [8]. Translation initiation of most mRNAs needs the assembly of eukaryotic initiation factor 4F (eIF4F) complex that can recruit ribosomes to the 5’‐terminus of mRNAs [9]. The eIF4F complex comprises three different proteins: the DEAD‐box helicase eIF4A, the scaffold protein eIF4G, and the cap‐binding protein eIF4E. Of these, eIF4E is the only one that can bind to the mRNAs cap structure directly and thus becomes an important limiting factor in controlling translation rate [10]. The rate‐limiting process regulated by eIF4E drives proteins’ selective expression. However, the overexpression of eIF4E improves the translation efficiency of numerous oncogenic proteins such as survivin and cyclin D1. These selective alterations of mRNA translation typically contribute to tumor cells growth and proliferation, tumor metastasis and therapeutic resistance [11, 12]. Moreover, it has been recognized that eIF4F‐dependent translational control can affect the tumor microenvironment (TME) and enhance cancer cell fitness. Meanwhile, the eIF4E axis inhibition can enhance antitumor immunity [13]. Importantly, the overexpression of p‐eIF4E is correlated positively with metastasis and poor prognosis in patients with NSCLC. These findings underscore the importance for leveraging targeted therapeutics against eIF4E in the clinical management of NSCLC [14, 15].

The mitogen‐activated protein kinase interacting kinases (MNKs) can phosphorylate eIF4E at serine 209 to initiate subsequent mRNA translation. MNKs exist as two different isoforms: MNK1 and MNK2, and both of them can phosphorylate their substrate eIF4E when MNKs and eIF4E are bound to the scaffold protein eIF4G [16]. Indeed, MNKs have been verified as the only kinases that phosphorylates eIF4E so far [17]. Notably, genetic knockout of MNK genes in murine models exhibits negligible impact on normal cellular functions or phenotypes, suggesting MNKs as potentially safe targets for anticancer therapy [18, 19]. Therapeutic targeting of MNK kinases holds promise for reshaping the TME and fostering T cells infiltration at tumor sites [20]. MNK inhibition can also sensitize the efficacy of chemotherapy for cancer therapy [21, 22].

Aberrant angiogenesis is an indispensable requirement for tumorigenesis and cancer progression. Within the angiogenic cascade, multiple signaling pathways orchestrate this process, with vascular endothelial growth factors (VEGFs) and their cognate receptors, VEGFRs, assuming paramount importance. The VEGFs/VEGFR axis represents one of the most important proangiogenic mediators [23]. Hitherto, a total of three VEGFRs have been identified: VEGFR1 is mainly responsible for hematopoietic cells development; VEGFR2 plays crucial roles in vascular endothelial cells growth and proliferation; and VEGFR3 participates in the development of vascular endothelial cells [24]. The excessive activation of VEGFR2 tends to promote endothelial cell proliferation, tube formation as well as increased vascular permeability [25]. After binding to VEGF, VEGFR2 becomes phosphorylated at some intracellular tyrosine residues, of which Tyr1175 phosphorylation is critical for activating downstream pathways. The inhibition of VEGFR2 has the capability to promote vascular normalization and alter the TME, which allows the infiltration of various immune cells to delay tumor progression. Thus, VEGFR2 has emerged as a potential anticancer target [23, 26].

Immunotherapy has emerged as a significant clinical breakthrough in the management of solid tumors recently, with immune checkpoint inhibitors (ICIs) gaining widespread adoption in clinical practice, particularly among patients with advanced‐stage NSCLC [27]. As the first‐line therapeutic strategy, platinum‐based chemotherapy and targeted therapy might further increase clinical efficacy. Identifying optimal combinations such as ICIs or chemotherapy plus targeted drugs is expected to provide more clinical benefits for NSCLC patients [28].

Based on the above findings, it is conceivable that targeting the MNK and VEGFR2 pathways presents a promising strategy, which would be complementary to current approaches for treating NSCLC. In the present study, we tested the antitumor efficacy of JDB153, an oral dual MNK/VEGR2 inhibitor and investigated its combinational effects with either anti‐PD‐1 antibody or cisplatin in lung cancer. The results showed that JDB153 exhibited effective inhibitory activity on lung cancer cells and the combined therapeutic strategy could delay tumor growth in mice tumor models markedly. This study explored the potential of JDB153 to treat patients with NSCLC and provided theoretical basis for the further application of JDB153 in combination with anti‐PD‐1 therapy or cisplatin in clinical practice.

## 2 Result

### 2.1 Elevated Expression of p‐eIF4E Correlated with Poor Prognosis of Lung Cancer Patients

**FIGURE 1:**
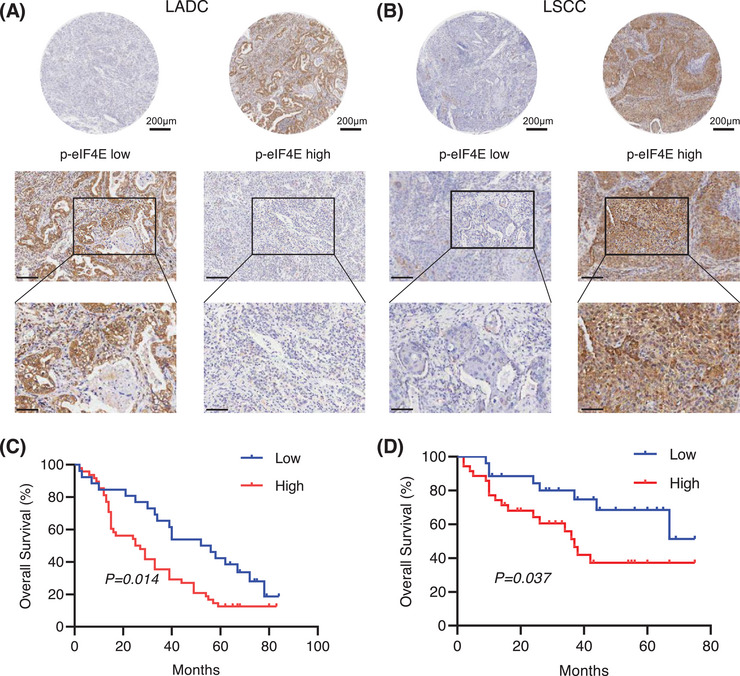
The expression of the p‐eIF4E in human NSCLC tumor tissues and its correlation with patient's prognosis. (A) and (B) Representative immunohistochemical staining of p‐eIF4E in human LADC and LSCC tissues. High and low suggested the expression level of p‐eIF4E in human tumor microarray. (C) Kaplan–Meier survival curves of 74 LADC patients. (D) Kaplan–Meier survival curves of 61 LSCC patients.

To investigate the association between p‐eIF4E and prognosis in human lung cancer, a total of 135 lung cancer patient's tumor tissues, including 74 lung adenocarcinoma (LADC) and 61 lung squamous carcinoma (LSCC), were collected and analyzed. The baseline characteristics of all enrolled patients and related comparisons were presented in Tables S1 and S2. The expression of p‐eIF4E protein was evaluated using immunohistochemical staining. Of the entire patients, 83 (61.5%) showed high expression and 52 (38.5%) had low expression of p‐eIF4E (Figure 1A,B). It was found that high expression of p‐eIF4E was closely related to reduced overall survival (OS) in LADC patients (Figure 1C; log‐rank *p *= 0.014; HR = 1.9, 95% CI = 1.15–3.15), by using Kaplan–Meier method. Similarly, in patients with LSCC, high expression of p‐eIF4E was significantly associated with shorter survival time (Figure 1D; log‐rank *p *= 0.037, HR = 2.32, 95% CI = 1.07–5.01). Overall, the above results indicated that elevated expression of p‐IF4E was correlated with poor prognosis in NSCLC patients and may act as an independent prognostic factor for patients with NSCLC.

### 2.2 JDB153 Inhibited the Viabilities and Colony Formation of Lung Cancer Cells in Vitro

**FIGURE 2:**
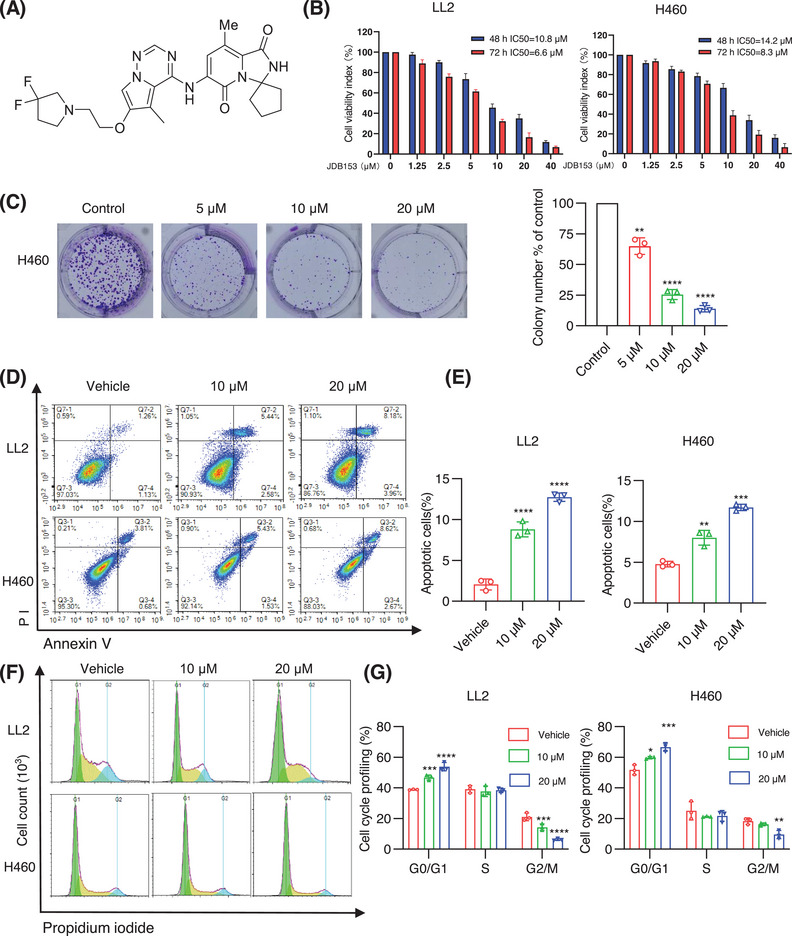
JDB153 inhibits cell viability and colony formation, induces cell apoptosis and cell cycle arrest of lung cancer cells. (A) The chemical structure of JDB153. (B) LL2 and H460 cells were treated with different concentrations of JDB153 for 48 and 72 h, and then cell viability was assessed by CCK‐8 assay. (C) Representative images of colony formation assay in H460 cell after drug treatment. (D) LL2 and H460 cells were treated with JDB153 for 48 h, stained with Annexin V/PI and cell apoptosis rates were determined through flow cytometry. (E) Quantitative analysis of apoptotic cell proportion. (F) The effects of JDB153 on cell cycle distribution of LL2 and H460 cells were detected by flow cytometry. (G) Quantification of the cell cycle distribution. *p < 0.05, **p < 0.01, ***p < 0.001, ****p < 0.0001 versus vehicle.

The chemical structure of JDB153 was shown in Figure 2A. There is increasing understanding that the activity of eIF4E is abnormally increased to stimulate cell growth, proliferation, and translation of related proteins in cancer initiation and development [29]. Consequently, to detect the influence of inhibiting eIF4E phosphorylation on lung cancer cells in vitro, we used the mouse lung cancer cell LL2 and the human NSCLC cell line H460 for in vitro experiments by Cell Counting Kit‐8 (CCK‐8) assay. As depicted in Figure 2B, JDB153 exhibited antiproliferative activity on lung cancer cell in a both dose‐dependent and time‐dependent manner. The 50% inhibitive concentrations (IC_50_) of JDB153 on LL2 cell and H460 cell for 48 h were 10.8 and 14.2 µM, and for 72 h were 6.6 and 8.3 µM, respectively. Colony formation was tested to assess the inhibitory effect of JDB153 on H460 cells. The data suggested that colony formation capacity of H460 cells was inhibited in a dose‐dependent manner (Figure 2C). When the drug concentration reached at 20 µM, the colony number was obviously decreased compared with the control.

### 2.3 JDB153 Induced Apoptosis and Cell Cycle Arrest of Lung Cancer Cells

The inactivation of apoptosis is a critical process for cancer initiation and development. The evasion of apoptosis is also a hallmark of cancer. To examine the effects of JDB153 on apoptosis in lung cancer cells, Annexin‐V FITC staining was used to determine the apoptosis extent. As shown in Figure 2D,E, the concentration‐dependent apoptosis in LL2 and H460 cells was observed after JDB153 treatment for 48 h. Specifically, the total proportion of early and late apoptotic LL2 cells was elevated from 2.39% (vehicle control) to 8.02 and 12.14% in response to 10 and 20 µM, respectively. Similarly, the total apoptotic H460 cells percentage was increased from 4.49% (vehicle control) to 6.96 and 11.29% after treatment of JDB153 in concentrations of 10 and 20 µM, respectively. The above data indicated that inducting cell apoptosis was also a potential mechanism by which JDB153 exerted its antitumor effects.

The dysregulation of cell cycle progression was an important hallmark of cancer. To further explore the possible mechanisms of the inhibitory effects of JDB153 on lung cancer cells, the changes of cell cycle distribution were evaluated. As shown in Figure 2F,G, following 20 µM JDB153 treatment for 48 h, the G0/G1 phase distribution of LL2 cells was obviously increased that of vehicle treatment, whereas the G2/M phase was decreased largely. Similar results were observed in H460 cells. All these data suggested that JDB153 could arrest lung cancer cells in the G0/G1 phase, which might contribute toward its anticancer effects.

### 2.4 JDB153 Suppressed the Migration and Invasion of Lung Cancer Cells

**FIGURE 3:**
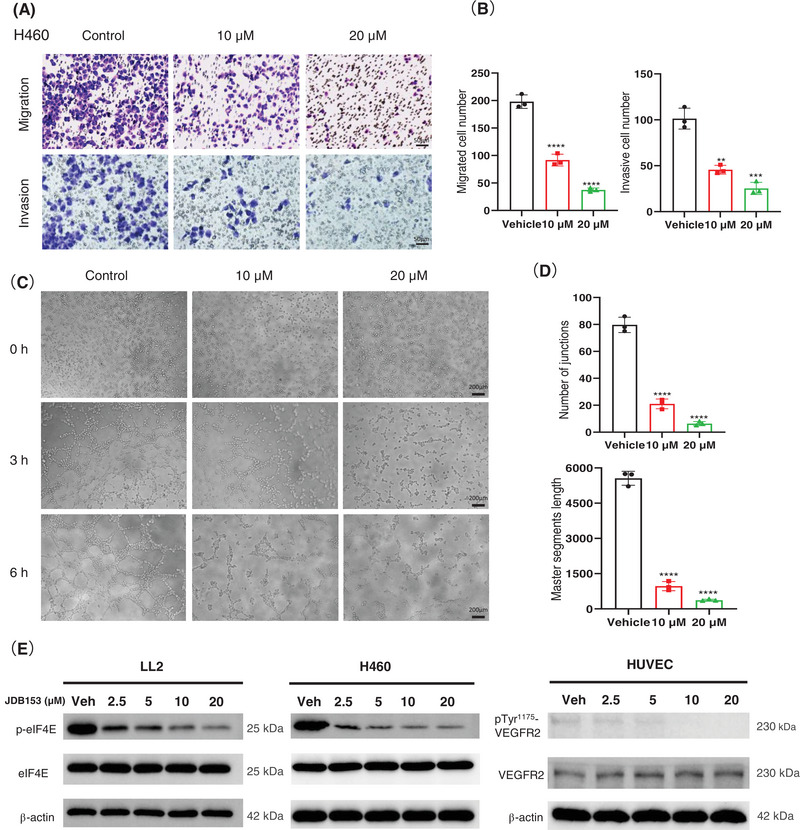
The inhibitory effects of JDB153 on cell migration, invasion, angiogenesis, and the phosphorylation of eIF4E and VEGFR2. (A) and (B) Cell migration and invasion capacity of H460 cell were tested by transwell assay following JDB153 treatment. (C) HUVEC was used to detect the inhibitory effect of JDB153 on angiogenesis. Representative images of tube formation assay. (D) Quantification of the angiogenesis in (C). (E) The phosphorylation of eIF4E was determined by Western blot analysis on LL2 and H460 cells treated with indicated concentrations of JDB153 for 48 h. The phosphorylation of VEGFR2 was detected on HUVEC after treating by indicated concentrations of JDB153 for 72 h. **p < 0.01, ***p < 0.001, ****p < 0.0001 versus vehicle.

Metastasis was one of the predominant contributors to mortality associated with lung cancer. The migratory and invasive capacity of cancer cell was vital to cancer progression and metastasis. Consequently, we performed transwell assays to evaluate the effect of JDB153 on H460 cells migratory and invasive ability. As suggested in Figure 3A,B, the migrated and invasive cell numbers following JDB153 treatment were obviously decreased compared with the vehicle group in a concentration‐dependent manner.

### 2.5 JDB153 Inhibited Angiogenesis and the Phosphorylation of eIF4E and VEGFR2 in Vitro

Abnormal angiogenesis is an indispensable requirement for tumorigenesis and cancer progression. Endothelial cells that had the proliferative and migratory capacity could form net structure spontaneously when they were stimulated by angiogenic signalings, and thereby they could simulate the angiogenesis process in vitro. To assess the suppressive role of JDB153 on angiogenesis, we conducted the tube formation assay. As demonstrated in Figure 3C, the tubular structure after 3 h of receiving JDB153 treatment with 10 or 20 µM was inhibited compared with the vehicle treatment. After 6 h of JDB153 treatment, the tube formation was dramatically inhibited by decreasing the number of junctions and master segment length dose dependently (Figure 3D), which suggested that JDB153 was able to inhibit angiogenesis in vitro.

We also wanted to explore whether JDB153 would influence eIF4E's phosphorylation in tumor cells. Western blot results revealed that JDB153 effectively suppressed eIF4E phosphorylation in LL2 and H460 cells in a concentration‐dependent manner (Figure 3E), but there was no significant change of eIF4E protein expression after JDB153 treatment with different concentrations. Meanwhile, given the fact that the phosphorylation of VEGFR2 could active its downstream protein kinase and then stimulate angiogenesis, we studied the effect of JDB153 on VEGFR2 phosphorylation at Tyr1175 that was indispensable for activating downstream pathway. As shown in Figure 3E, JDB153 clearly reduced the phosphorylation of VEGFR2 at Tyr1175 in human umbilical vein endothelial cells (HUVECs) in a concentration‐dependent manner, whereas the total level of VEGFR2 was not influenced by JDB153.

### 2.6 JDB153 Potentiated the Antitumor Activity of Immunotherapy in LL2 Subcutaneous Tumor Model

**FIGURE 4:**
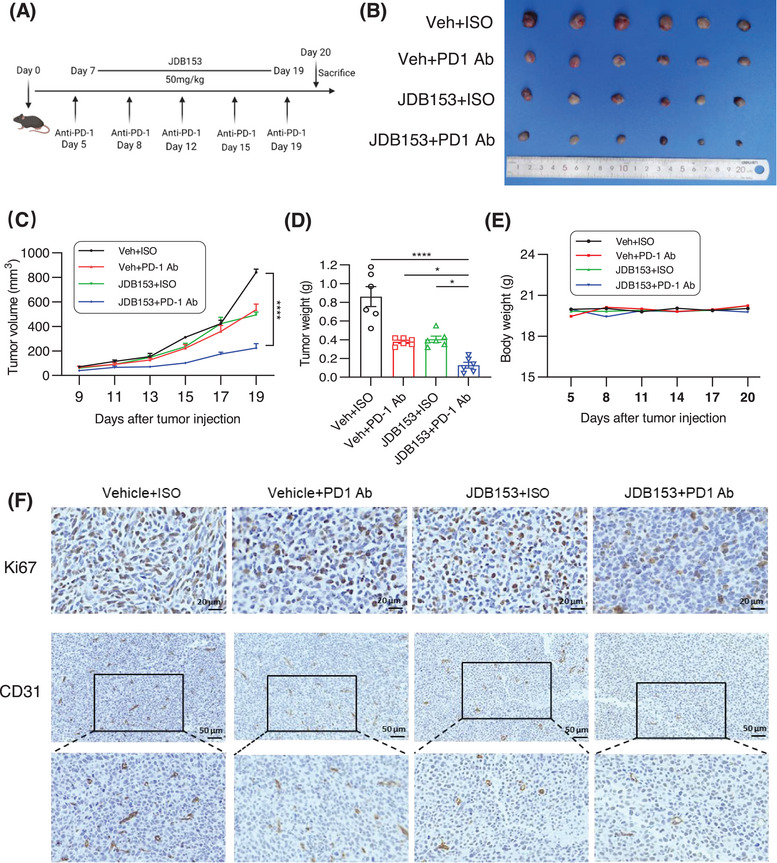
The synergistic antitumor effect of JDB153 and PD‐1 Ab in LL2 subcutaneous tumor model. (A) Therapy regimen of the experiment. (B) The gross specimens of subcutaneous LL2 tumors at the endpoint of this experiment. (C) and (D) Tumor growth curves and tumor weight at the sacrifice of subcutaneous model. Data were shown as mean ± SD. (E) Body weight changes of mice. (F) The expression of Ki67 and CD31 was detected by IHC in LL2 tumor tissues treated with JDB153 and PD‐1 Ab. Scale bar = 20 mm or 50 mm. *p < 0.05, **p < 0.01, ***p < 0.001, ****p < 0.0001.

Immunotherapy has revolutionized cancer treatment recently and represents a favorable method in advanced solid tumors such as NSCLC. Given the inhibitory effect of JDB153 on LL2 cell growth and proliferation, we established a subcutaneous tumor model using LL2 cell to assess the therapeutic effect of JDB153 alone or in combination with anti‐PD1 antibody (PD‐1 Ab). Mice‐bearing tumors were randomized into four different groups and the treatment regimen was shown in Figure 4A. The PD‐1 Ab was administered twice a week intraperitoneally (10 mg/kg) and JDB153 was administered orally daily (50 mg/kg). As depicted in Figure 4B,C, after treatment for 14 days, the growth of tumors in mice treated with JDB153 or PD‐1 Ab was delayed compared with the vehicle group. Additionally, combinational treatment with JDB153 and PD‐1 Ab further inhibited tumor growth compared with either treatment alone. Analogously, the tumor weight of mice treated by coadministration of JDB153 and PD‐1 Ab was obviously less than that of mice only receiving JDB153 or PD‐1 Ab (Figure 4D). Moreover, to preliminary estimate the side effects of JDB153 and PD‐1 Ab, the body weights of mice were measured and the result showed no obvious difference in mouse weight among each group (Figure 4E).

To further evaluate tumor cell proliferation in vivo characterized by Ki67‐positive rates in tumor tissues, immunohistochemical analyses were performed. As depicted in Figure 4F, the expression of Ki67 in the JDB153 and PD‐1 Ab group was reduced than that in vehicle group, and the Ki67 expression in the combinational treatment group was the lowest. In addition, we sought to evaluate the effect of JDB153 on tumor angiogenesis by performing IHC using the angiogenesis marker CD31. Consistent with the finding that JDB153 could inhibit neovascularization in vitro, JDB153 also led to decreased expression of CD31 compared with that of vehicle. Meanwhile, JDB153 plus PD‐1 Ab treatment also exhibited remarkable antiangiogenesis efficacy compared with those of either JDB153 or PD‐1 Ab monotherapy.

### 2.7 JDB153 Enhanced Immunotherapy Efficiency by Affecting Immune Cells Infiltration in the TME

**FIGURE 5:**
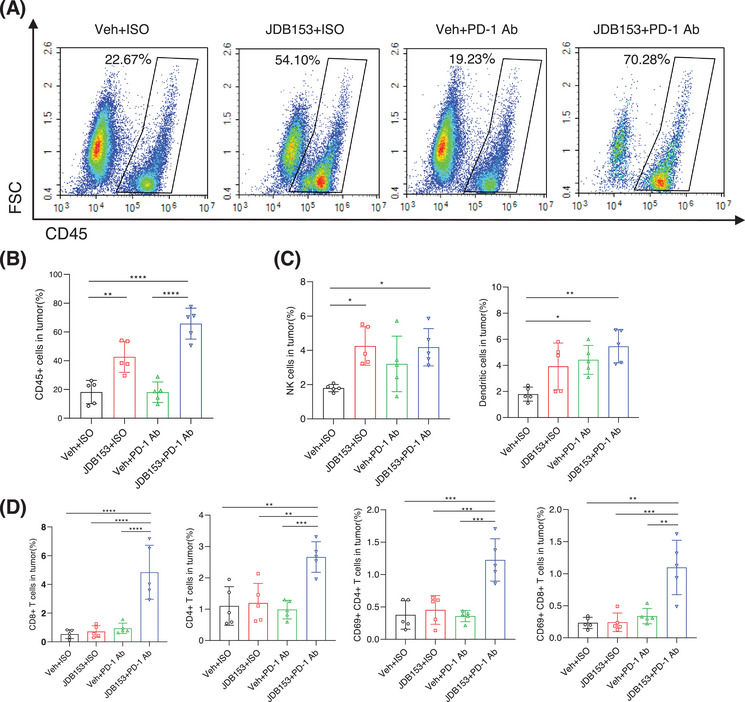
The effects of JDB153 in combination with PD‐1 Ab on the TME. (A) and (B) The combined therapy significantly increased the proportion of CD45+ tumor‐infiltrating cells compared with the group of monotherapy or vehicle treatment. (C) The implications of JDB153 and PD‐1 Ab treatment on DCs and NK cells. (D)The application of JDB153 plus PD‐1 Ab promoted antitumor immunity by enhancing the infiltration of CD4+ T cell, CD8+ T cell, activated CD4+ T cell, and activated CD8+ T cell. Data were expressed as mean ± SD. *p < 0.05, **p < 0.01, ***p < 0.001, ****p < 0.0001.

To further investigate the underlying mechanisms by which JDB153 increased the response to anti‐PD‐1 therapy, we detected the proportions of tumor‐infiltrated immune cells in the TME via flow cytometry. To our surprise, the application of JDB153 plus PD‐1 Ab significantly increased the proportion of CD45+ tumor‐infiltrating cells composed of CD4+ and CD8+ cells compared with the group of monotherapy or vehicle treatment (Figure 5A,B). Based on the above discovery, we then analyzed the changes of CD4+ and CD8+ T cells in the whole TME. The results showed that the percentage of CD4+ T cell and CD8+ T cell in JDB153 plus PD‐1 Ab group were markedly enhanced than that of monotherapy or vehicle group, which suggested that the combinational therapeutic regimen effectively aroused antitumor immune. Analogously, activated CD4+ T cell and CD8+ T cell characterized by the expression of CD69 were increased in the TME following the treatment of JDB153 plus PD‐1 Ab (Figure 5D). These results collectively indicated that JDB153 could improve the antitumor immune of T cells in the TME.

Natural killer (NK) cells play an integral role in anticancer immunity because of their ability to identify and eliminate tumor cells [30]. We thus investigated whether the MNK inhibition could change NK cells distribution within the TME. As a result (Figure 5C), JDB153 treatment promoted NK cells infiltration in tumor tissues compared with the group of PD‐1 Ab therapy and vehicle. Despite NK cells population was also increased following PD‐1 Ab plus JDB153 therapy, there were no significant changes between this combinational treatment and JBD153 monotherapy. As such, we speculated that the elevation in the percentages of NK cells might be attributed to the application of JDB153. Apart from NK cells, dendritic cells (DCs) could promote anticancer T‐cell response by cross‐presenting tumor‐associated antigens to T cells and were considered as central components of the TME [31]. Since the observation that JDB153 could induce tumor cell death in vitro, we hypothesized that JDB153 would also result in the activation of APCs within the TME to fortify anticancer responses. We then analyzed the changes of infiltrating CD11c+ DC cells in the TME among the different treatment groups. As shown in Figure 5C, both of JDB153 and PD‐1 Ab treatment promoted DCs infiltration into the TME, but no significant differences of DCs percentages were found between the JDB153 and the vehicle group. The elevation of DCs within the TME might be primarily ascribed to the treatment of PD‐1 Ab.

### 2.8 JDB153 Suppressed Tumor Growth and Potentiated the Antitumor Activities of Chemotherapy in NSCLC Xenograft Model

**FIGURE 6:**
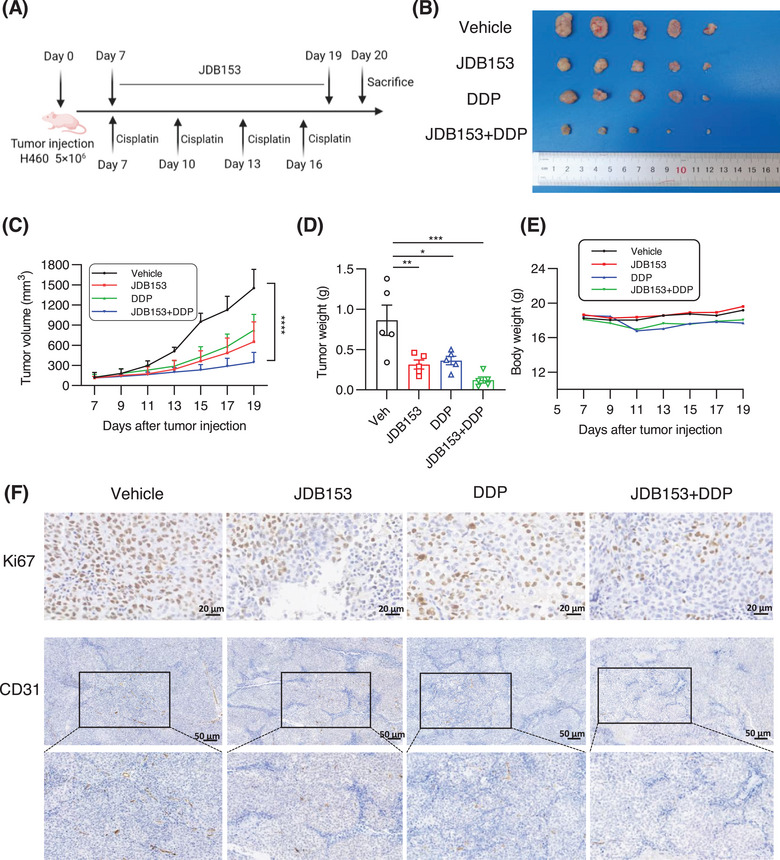
The synergistic antitumor effect of JDB153 and cisplatin in the H460 xenograft model. (A) Therapy regimen of the experiment. (B) The gross specimens of subcutaneous H460 tumors at the endpoint of experiment. (C) and (D) Tumor growth curves and tumor weight at the sacrifice of subcutaneous xenograft model. Data were shown as mean ± SD. (E) Body weight changes of mice. (F) The expression of Ki67 and CD31 was detected by IHC in H460 xenograft tissues treated with JDB153 and cisplatin. Scale bar = 20 or 50 mm. *p < 0.05, **p < 0.01, ***p < 0.001.

In addition to immunotherapy, platinum‐based doublet chemotherapy was also an effective therapeutic option for patients with lung cancer, especially for NSCLC patients. The inhibition of MNK could suppress H460 cell's growth, migration, and invasion. Thus, we evaluated whether JDB153 and cisplatin interact synergistically in inhibiting NSCLC growth using H460 xenograft model. BALB/c nude mice were subcutaneously implanted with approximately 5 × 10^6^ H460 cells. The cisplatin (DDP) was administered intraperitoneally at the dose of 2 mg/kg twice a week and JDB153 was administered orally daily (50 mg/kg) after 1 week of tumor cells injection (Figure 6A). After treatment for 14 days, the tumor volume in mice treated with JDB153 or in combination with DDP was lower than that of mice in the vehicle group. Meanwhile, combinational treatment with JDB153 and DDP also delayed tumor growth compared with either treatment alone (Figure 6B,C). Similarly, tumor weight of combined treatment group was less than that of mice receiving monotherapy group and the vehicle group (Figure 6D). Additionally, despite the body weight of DDP group's mice and combined group's mice was decreased, there were no significant statistic difference compared with the JDB153 or vehicle group (Figure 6E).

Immunohistochemical analyses were performed to evaluate tumor cell proliferation in vivo, characterized by the expression of Ki67. As a result (Figure 6F), the single use of JDB153 and DDP decreased Ki67 expression in tumor tissues compared with the vehicle group. Moreover, the Ki67 expression of concomitant application of JDB153 and DDP was the lowest relative to either treatment alone and the vehicle group. Similar results were also reflected in the expression of CD31 in tumor tissues, an angiogenesis marker, which indicated that the concomitant use of JDB153 and DDP synergistically inhibited neovascularization.

### 2.9 JDB153 in Combination With PD‐1 Ab or Cisplatin Shows Reliable Safety in Vivo

As mentioned above, there were no notable alterations in body weight observed between mice treated with the vehicle and JDB153, which suggested that the usage of JDB153 may exhibit a favorable safety profile in vivo. To further evaluate the in vivo safety of JDB153 alone or in combination with PD‐1 Ab therapy or cisplatin, we conducted H&E staining so as to analyze whether there were pathological changes in the major organs including heart, liver, spleen, lung, and kidney. No obvious pathologic changes and adverse effects were observed in JDB153 monotherapy or combinational therapy group (Figures S1A and S2A). Moreover, we also measured the changes in blood biochemistry following treatment. The main monitoring indexes included TP, ALB, ALT, AST, CREA, UREA, UA, LDL, LDH, and CK‐MB. Despite some fluctuations among different groups were observed, all of the changes ranged within normal values (Figures S1B and S2B), which indicated JDB153 as monotherapy or in combination with immunotherapy or chemotherapy is relatively safe and well tolerated.

## 3 Discussion

Lung cancer persists as the predominant etiology of cancer‐related fatality worldwide and NSCLC is the main type. Diagnosis typically occurs at an advanced disease stage for the majority of NSCLC patients, rendering surgical interventions often impracticable. However, the widespread utilization of chemotherapy in clinical settings is frequently impeded by unforeseen drug resistance and suboptimal patient responses, thereby curtailing the further utilization of platinum‐based agents [32]. Recent advancements in mechanism‐driven targeted therapy and immunotherapy have markedly reshaped the therapeutic landscape of NSCLC. Searching for effectively combined therapeutic regime is a major focus of current cancer research.

The components of translation machinery integrate nearly all oncogenic signals. Recent advancements of in‐depth understanding the aberrant translation control process led to the identification of diverse therapeutic targets for cancer treatment, and among all the eIF4E represents a promising one due to its multiple roles in carcinogenesis and progression. Accumulating evidence has shown that the overexpression of eIF4E and its phosphorylated form is correlated positively with tumor burden and also leads to poor prognosis in NSCLC [33, 34]. MNKs are the only kinases to phosphorylate eIF4E. Clinically, the expression of MNK can predict poor prognosis in NSCLC. However, the knockout of MNK gene do not affect normal cells function in mice, which suggests that targeting MNK may be an ideal therapeutic approach to selectively target cancer cells and motivates the development of drug candidates targeting MNK for cancer therapy. At present, some MNK inhibitors have been designed and tested in both preclinical study and clinical trial. These drugs mainly include natural products and their derivatives and synthesized compounds. For instance, eFT508 is a novel inhibitor that shows potent activity against MNK1 and MNK2. Several ongoing clinical trials evaluate its therapeutic efficiency for solid tumors and hematological malignancies as monotherapy or in combination with anti‐PD‐1 drugs. Furthermore, ETC‐206 is a small molecule with selective inhibition of enzymatic activity of MNK 1/2 kinases. Although the majority of ETC‐206 are at the preclinical stages, some of them are being tested in clinical settings. The area of study targeting MNK for cancer treatment still needs additional exploration.

The inhibition of tumor angiogenesis is also a promising approach to delay cancer progression. As a crucial signal for initiating angiogenesis, VEGFR2 has emerged and been extensively tested in clinical trials for cancer treatment. In this context, we use a novel MNK/VEGFR2 dual inhibitor named JDB153 and provide available data to demonstrate its antitumor effect in combination with chemotherapy or immunotherapy, with the aim to aid feasible optimization for NSCLC treatment.

In the present study, our results revealed that p‐eIF4E was overexpressed in both LADC and LSCC tissues and associated with poor OS of patients with lung cancer, which was in agreement with previous literature [14, 34]. The above findings suggested that targeting eIF4E would be a feasible strategy to improve the outcomes of patients with NSCLC. We then demonstrated the anticancer effects of JDB153 in mouse Lewis lung carcinoma cell and human NSCLC cell H460 and revealed underlying mechanisms. First, we discovered that JDB153 could inhibit cell proliferation of LL2 and H460 in a dose‐dependent way. JDB153 also repressed the colony formation capacity of H460 cell. Moreover, aberrant cell apoptosis and cell cycle usually contributed to cancer initiation and progression. In this context, JDB153 was found to promote apoptotic cell death and induce cell cycle arrest in G0/G1 phase of lung cancer cells in vitro dose dependently. Also, JDB153 could inhibit angiogenesis and the phosphorylation of eIF4E and VEGFR2 in a concentration‐dependent manner in vitro obviously.

Based on the above results, we further explored the antitumor effects and possible mechanisms of JDB153 in vivo. We constructed LL2 lung cancer subcutaneous model and H460 NSCLC xenograft model in mice to evaluate combinational therapy outcomes of JDB153 plus immunotherapy (PD‐1 Ab) and chemotherapy (cisplatin) respectively. As a result, the growth of tumors in mice treated with combined therapy was markedly delayed compared with the vehicle group or monotherapy groups. A recent study summarized the critical roles of eIF4F‐driven mRNA translation in regulating the TME [13], and therefore, we researched the specific influences of p‐eIF4F inhibition on the TME in LL2 subcutaneous model. Somewhat surprisingly, we observed the markedly elevated proportion of CD45+ tumor‐infiltrating cells following the application of JDB153 compared with the vehicle group. On this basis, we detected the changes of immune cells in the whole TME and the results showed that MNK inhibition by JDB153 plus PD‐1 Ab therapy contributed to the infiltration of CD4+ and CD8+ T cell, activated CD4+, CD8+ T cells, NK cells, and DCs, which could promote antitumor immune. Moreover, immunohistochemical results of tumor tissue showed that the expression of Ki67 and CD31 of combinational therapy group was reduced significantly compared with the vehicle and monotherapy groups. Thus, we hypothesized that suppressing tumor angiogenesis affected by JDB153 was another way to hinder carcinogenic process.

In the last few years, immunotherapy has rapidly transformed the anticancer treatment and drug development landscape. The emergence of antibodies targeting immune checkpoints exploited by tumors for growth and metastasis is a landmark and continues to accelerate. Simultaneously, inherent and acquired resistance following chemotherapy results in the design and development of targeted drugs. Previous studies demonstrated that the MNK–eIF4E axis supported tumor metastasis and immune suppression, and MNK inhibition impaired protumor phenotype switching and potentiated antitumor immune responses in melanoma and breast cancer [35, 36]. Nevertheless, little is known about the affection of MNK–eIF4E axis inhibition on lung cancer growth and its TME. The results of our study proved the influence of inhibiting MNK on lung cancer development and highlighted the significance of JDB153 in combination with PD‐1 Ab, which was expected to provide more effective and practicable therapeutic options for NSCLC treatment.

We also explored the combinational antitumor effects of JDB153 plus cisplatin using H460 xenograft model. The combinational treatment delayed tumor growth compared with either treatment alone markedly. This regimen also reduced the expression levels of proliferation marker Ki‐67 and angiogenesis marker CD31 in tumor tissues significantly. More importantly, the above regimen demonstrated good in vivo safety characteristics, as evidenced by the absence of significant treatment‐related adverse effects in vital organ systems, which further supported that JDB153 could be used as a promising target for NSCLC.

There are several limitations of this study. For instance, our research focused on elucidating the inhibitory effects of JDB153 on tumor growth and the TME but largely relied on established lung cancer cell lines and xenograft animal models. As such, using primary lung cancer cell derived from patients may yield more convincing results. Furthermore, we observed precipitation when a high concentration of the JDB153 was dissolved in the culture medium in vitro experiments. Therefore, we employed 0.5‰ Tween 80 as a solubilizer to enhance drug solubility. In this condition, the impact of Tween 80 on the in vitro experiment results should be taken into account. Additionally, searching for the best administration regimens and legitimate sequence holds promise for enhancing the inhibitory effects on NSCLC when combining JDB153 with immunotherapy or chemotherapy.

In summary, JDB153, a novel dual MNK/VEGFR2 inhibitor, exhibits effective therapeutic activity against NSCLC with favorable safety. It inhibits the phosphorylation of eIF4E and VEGFR2 dramatically and suppresses the growth and proliferation of both LL2 and H460 cells. JDB153 can promote cell apoptosis, induce cell cycle arrest, inhibit tumor neovascularization, as well as hinder cancer cell migration and invasion. In the experiments in vivo, JDB153 in combination with PD‐1 Ab or cisplatin exhibits synergetic antitumor capacity with well tolerance. Thus, it is necessary to assess the antitumor effects of JDB153 as monotherapy or in combination with immunotherapy or chemotherapy for NSCLC in clinical settings, which would be complementary to current antitumor approaches and aid the optimization of NSCLC treatment.

## 4 Materials And Methods

### 4.1 Reagents and Cell Culture

We designed a series of pyrrolotriazine compounds to identify highly active and selective MNK and VEGFR2 inhibitors. Through rigorous screening, we finally found that JDB153 exhibited potent inhibitory activity against MNK and VEGFR2. The kinase inhibitory activities of JDB153 were assessed using a radiometric kinase assay. The IC_50_ value of JDB153 on MNK1 and MNK2 were 26.6 and 17 nM, respectively. The JDB153 exhibited obvious inhibitory effect of VEGFR2, with the IC_50_ value of 3 nM.

The mouse lung cancer cell LL2, human NSCLC cell H460 and HUVECs were obtained from the American Type Culture Collection (ATCC, Rockville, MD, USA). LL2 was cultured in Dulbecco's modified Eagle's medium (DMEM) containing 10% fetal bovine serum (FBS) and 1% antibiotics (penicillin and streptomycin). H460 was cultured in RPIM 1640 supplemented with 10% FBS and 1% antibiotics. HUVECs were cultured in DMEM/F12 medium containing FBS and antibiotics. All the cells were maintained at 37°C in a humidified atmosphere with 5% CO_2_. The CCK‐8 was purchased from MedChem Express. The FITC‐Annexin V apoptosis detection kit and propidium iodide (PI) were purchased from BD Biosciences. The RIPA buffer, proteinase inhibitor, and phosphatase inhibitor were obtained from Beyotime.

### 4.2 Animals

The experimental animals used in this study were female C57BL/6 wild type mice (6–8 weeks old) and female BALB/c nude mice (6–8 weeks old) provided by HFK Biotechnology Company. All of the animals were housed under the specific‐pathogen‐free condition and maintained at 21°C and 55% humidity in an animal facility. All the protocols of the study were approved by the Institutional Animal Care and Use Committee of Sichuan University (Chengdu, Sichuan, China).

### 4.3 Tumor Models Establishment and Treatments

Before the injection of cancer cells, all the mice were permitted to adapt the circumstance for 1 week. To explore the combinational therapeutic effect of JDB153 plus anti‐PD‐1 treatment, approximately 5 × 10^5^ LL2 cells were suspended in 100 µL medium without FBS and antibiotics and were subcutaneously implanted into female C57BL/6 mice. When the tumor volumes reached about 50–100 mm^3^, mice were assigned to four different groups randomly and treated with the following agents (1) vehicle plus isotype‐matched IgG antibody (ISO; BioXCell); (2) JDB153 plus ISO; (3) vehicle plus anti‐mouse PD‐1 antibody (αPD‐1 (CD279); BioXCell); (4) JDB153 plus anti‐mouse PD‐1 antibody. The vehicle or JDB153 were administered orally daily and the dose of JDB153 was 50 mg/kg. The ISO and anti PD‐1 antibody were administered twice a week intraperitoneally (10 mg/kg) until the end of the study. To investigate the combinational therapeutic efficacy of JDB153 plus cisplatin in NSCLC, a xenograft model was established. Approximately 5 × 10^6^ H460 cells were subcutaneously injected into the right posterior flanks of female BALB/c nude mice. Analogously, while the tumor volumes reached about 50–100 mm^3^, mice were assigned randomly to each group and treated with the following agents (1) single vehicle; (2) single JDB153; (3) single cisplatin (MedChemExpress); (4) JDB153 plus cisplatin. The cisplatin was administered intraperitoneally at the dose of 2 mg/kg twice a week. During the treatment period, the body weight and tumor volume were monitored and recorded every 2 days. The tumor volume was measured by two‐dimensional electronic caliper and calculated and then calculated according to the formula: volume (mm^3^) = length (mm) × width (mm)^2^  × 0.5. At the end of treatment, animals were sacrificed by cervical dislocation. Subsequently, the subcutaneous tumors were isolated and weighed, and the heart, liver, spleen, lung, and kidney of each mouse were also harvested. The tumor tissues and vital organs of mice were fixed with 4% formalin for further histopathology analysis.

### 4.4 Statistical Analyses

GraphPad Prism 9 software was used for statistical analysis. Results were represented as the mean ± standard deviation (SD). Survival was analyzed using Kaplan–Meier method. Comparisons between two groups were analyzed by Student's *t*‐test. Differences among multiple groups were analyzed by ANOVA analysis. *p* value < 0.05 was considered statistically significant. Other method details are provided in the Supporting Information section.

## Author Contributions

Xiawei Wei and Li Yang designed the study, provided conceptual idea and revised the manuscript. Maosen Xu, Tao Zhang, and Li Xu conducted experiments and analyzed data. Maosen Xu wrote the manuscript. Xue Li, Ziqi Zhang, Ruolan Xia, and Ning Jiang helped and guided experiments and critically revised the manuscript. All authors have read and approved the final version of the manuscript.

## Ethics Statement

The tissue specimens of patients with NSCLC and related study were approved by the Clinical Research Ethics Committee, Outdo BioTech (permit number: SHYJS‐CP‐1910013 and SHYJS‐CP‐1904014). Besides, all animal studies followed and approved by the Institutional Animal Care and Use Committee of Sichuan University (Chengdu, Sichuan, China) with the approval number 20230307041.

## Conflicts of Interest

Authors Xiawei Wei and Ning Jiang are employees in Jumbo Drug Co., Ltd., but have no potential relevant financial or nonfinancial interests to disclose. The other authors have no conflicts of interest to declare.

## Supporting information



Supporting Information

## Data Availability

The data included in this study are available upon request from the corresponding author.
