# Supplementary material for: Dual MNK/VEGFR2 Inhibitor JDB153 Enhances Immunotherapeutic Efficiency and Chemosensitivity in Lung Cancer
Source: MedComm (2020). 2025 Apr 29;6(5):e70155. doi: 10.1002/mco2.70155 (PMC12041661; doi:10.1002/mco2.70155)
Supplement: Supplementary file 1 — Supporting Information [file MCO2-6-e70155-s001.docx]

**Dual MNK/VEGFR2 inhibitor JDB153 enhances immunotherapeutic efficiency and chemosensitivity in lung cancer**

Maosen Xu^1#^, Li Xu^1#^, Tao Zhang^1#^, Xue Li^1^, Ziqi Zhang^1^, Ruolan Xia^1^, Ning Jiang^2^, Li Yang^1*^, Xiawei Wei^1,2*^

1. Laboratory of Aging Research and Cancer Drug Target, State Key Laboratory of Biotherapy, National Clinical Research Center for Geriatrics, West China Hospital, Sichuan University, No. 17, Block 3, Southern Renmin Road, Chengdu, Sichuan 610041, PR China.
2. Jumbo Drug Bank Co., Ltd., High-TechZone, Chengdu 610041, China.

^#^These authors contributed equally to the work.

* Corresponding author:

Li Yang: [yl.tracy73@gmail.com](mailto:yl.tracy73@gmail.com),

Xiawei Wei: [xiaweiwei@scu.edu.cn](mailto:xiaweiwei@scu.edu.cn)

**Supplemental Methods**

**Patient cohorts and tissue specimens**

The patient’s clinical data and related tumor specimens were provided by Shanghai Outdo Biotech (National Engineering Centre for Biochip at Shanghai, China). Tumor tissue specimens were obtained from 135 NSCLC patients who underwent surgical resections between May 2008 and September 2016. Clinic-pathological classifications were conducted according to the 8th edition of lung cancer classification. The overall survival (OS) is defined as the interval between definitive surgery and either death or the last observation taken and was used to evaluate patients’ prognosis in the current study. The histochemical score was assessed to establish the percentage of stained cells and the staining intensity referring to the previous studies ^1, 2^. Analyses and scoring were conducted by two independent pathologists who were blinded to patient’s diagnosis and their clinical outcomes. Briefly, distribution was scored as 0 (0%), 1(1~50%) as well as 2 (51~100%), which aimed to illustrate the percentage of positive cells in a single core. The signal intensity was scored as 0 (negative), 1 (weak), 2 (medium) as well as 3 (strong). Final expression scores were recorded as the summation of distribution score and intensity score. The final score 0, 1 and 2 indicated low expression whereas 3, 4, and 5 were considered as high expression. Kaplan-Meier survival analysis was leveraged to determine the relationship between the expression of p-eIF4E and patients’ survival.

As for the comparisons of baseline information between patients enrolled in the study, SPSS software (IBM, Version 20.0) was used to conduct statistical analyses. Briefly, descriptive statistics are described as median (±SD) for continuous variable and frequency (%) for categorical variables. Pearson’s chi-square or Fisher’s exact test was used to compare differences for categorical characteristics. The independent sample t-test was performed for continuous factors with normal distribution, whereas the Mann-Whitney U test was used for continuous factors without normal distribution. Bonferroni-adjusted significance tests were applied for pairwise comparisons. The statistical tests were two-sides; with statistical significance indicated by *P* values lower than 0.05.

**Cell proliferation assay**

Cell proliferation of LL2 and H460 was measured by the Counting Kit-8 (CCK-8), following the producer’s instructions. Briefly, LL2 and H460 cells underwent seeding process in a 96-well plate at 1~4 × 10^3^ cells/well density and were permitted to grow overnight to adhere to the wall. Then, 100 μl of medium containing the required concentration of JDB153 was added to each well for 48h or 72h. After incubation for given length of time, 90 μl fresh medium together with 10 μl CCK-8 solution were added into each well and the culture plate subsequently underwent incubation for 1~4 hours at 37°C. The absorbance of plates was detected at 450 nm by a microplate reader.

**Colony formation assay**

To evaluate the clonogenic capacity, approximately 500 cells/well of H460 were seeded in 6-well plates and then incubated overnight. Fresh medium containing indicated doses of JDB153 were added to each well and the cells were permitted to grow continuously. After the incubation for 10~14 days, wells were rinsed by PBS for 3 times. Then, cell colonies of each well were fixed with 4% polyoxymethylene for 10 min and were stained with 0.5% crystal violet for 10 min. Subsequently, cell colonies were washed 3 times with PBS and dried at room temperature. Finally, colonies containing more than 50 cells were photographed under light microscopy and counted to assess cells’ clonogenic capacity.

**Cell apoptosis assay**

For the apoptosis assay, cells were seeded in 12-well plates at the density of 5×10^4^ per well. JDB153 was added to each well at the appropriate concentrations for 48 h. Meanwhile, the DMSO-treat group was used as the control. After incubation at 37°C for 48 hours, the cells and supernatants were harvested and cleaned 2 times with ice-cold PBS. Then, according to the manufacturer’s instructions, the samples were incubated with Annexin V-FITC for 10 min and were labeled with propidium iodide (PI) for 5 min in the dark. The NovoExpress software was used to analyze cells’ apoptosis levels. Each experiment of different concentration treatment was repeated 3 times.

**Transwell assays**

To detect the effect of JDB 153 on the migration and invasion of H460 cells, the transwell assays were conducted using the 8μm pore-size transwell plates. In brief, H460 cells were plated in 200μL serum-free medium with different concentrations of JDB153 (0, 10, 20µM) on upper chambers inserted into a 24-well plate (5×10^4^ cells/well), and bottom chambers were filled with 600μL of medium containing 20% fetal bovine serum. After 48 hours of cell migration, the cells on the lower surface of the chamber were fixed by 4% paraformaldehyde and then were stained with 0.5% crystal violet. Meantime, cells on the upper chamber were removed with a cotton swab and migratory cells were counted and photographed. For cell invasion assessment, the upper chamber was precoated with diluted matrigel and cells were seeded into the chamber as previously conducted and cultured with 200μL serum-free medium containing different concentrations of JDB153 when the matrigel polymerization was achieved. After an incubation for 48 hours, the ompartments were removed and cleaned. Subsequently, the cells on the upper chamber were removed by a cotton swab and the cells on the lower surface of chamber were washed with PBS, fixed using 4% paraformaldehyde and stained by 0.5% crystal violet. The invasion cells were counted and photographed under a light microscope (Olympus Corporation). Each experiment was repeated at least three times.

**Tube formation assay**

The tube formation assay was performed as described previously^3^. Briefly, the precooled 96-well plate was coated with 50μL Matrigel per well. The HUVEC underwent starved treatment using a medium without FBS for 24 hours before the experiment. Then HUVEC was seeded into the Matrigel-coated 96-well plate at the density of 2 ×10^4^ cells per well and was cultivated by medium containing indicated doses of JDB153. Pictures were taken with the light microscope after JDB153 treatment for 0, 3 and 6h respectively. The ImageJ software was used to measure the number of junctions and master segment length.

**Western blot analysis**

The LL2 and H460 cell were treated with JDB153 for 48h and the HUVECs were treated with JDB153 for 72h. Then, all the above cells were washed with ice-cold PBS and then lysed in 1× RIPA lysis buffer that was supplemented with phosphatase inhibitor cocktail and protease inhibitor cocktail. Then, the cell lysates were collected and homogenized using an ultrasonic disruptor. The insoluble debris was removed by centrifugation at 13,000 rpm at 4 ^◦^C for 15 min. The protein concentrations were ascertained to ensure equal loading by the BCA Protein Assay Kit. Meanwhile, the protein samples were prepared with 5× loading buffer and were denatured by boiling at 100 ^◦^C for 5 min. The total protein of each sample was run through 12.5% or 7.5% sodium dodecyl sulphate-polyacrylamide gel electrophoresis (SDS-PAGE) gels and then transferred onto polyvinylidene difluoride (PVDF) membranes. After electrophoresis, the separated samples were transferred to 0.2μm or 0.45μm PVDF membranes and blocked with 5% milk dissolved in TBST for 1 h at room temperature. As for the detection of phosphorylated proteins, the PVDF membranes were blocked with 5% bovine serum albumin diluted in TBST for 1 h at room temperature. Subsequently, the PVDF membranes were washed for 3 times × 5 min and then were incubated overnight at 4°C with specific primary antibodies. When 3 times of washing with TBST was finished, the membranes underwent incubation for 1 hour with indicated HRP-coupled secondary antibodies at room temperature. The immunoreactive protein bands were imaged. Antibodies for western blot analysis were purchased from Abcam (eIF4E, ab33766, 1:1000), Huabio (p-eIF4E, ET1608-66, 1:1000), Cell Signaling Technology (VEGFR2, 2479, 1:1000; p-VEGFR2, 2478, 1:1000) and Santa Cruz (β-actin, 47778, 1:1000).

**Flow cytometry analysis**

Mice were euthanized for tumor tissue processing. Briefly, the tumor tissues were dissected and then cut into small pieces and digested by 10 ml RPMI-1640 medium buffer containing 0.5 mg/mL collagenase IV, 1 mg/mL collagenase I and 40U/mL Dnase I for 1h at 37°C. Subsequently, the suspensions were treated with red blood cell lysis buffer to lyse red blood cells. The digested cells were washed by PBS for 3 times and then dispersed in 100 ul PBS for staining by 1 ul fluorescence-conjugated antibodies for 30 min at 4°C. The above cells were then washed 2 times for flow cytometry analysis. Antibodies used for flow cytometry analysis were listed as follows: CD45 FITC (BioLegend, 103108), CD3-PerCP-Cy5.5 (BioLegend, 100328), CD4-BV421 (BioLegend, 100443), CD8-APC (BioLegend, 100712), CD69-PE/Cyanine7 (BioLegend, 104512), NK1.1-BV 650 (BioLegend, 108736), CD45-BV650 (BioLegend, 103151), CD11b-FITC (BioLegend, 101206), CD11c-APC (BioLegend, 117310). Data acquisition was performed by NovoCyte Flow Cytometerdata and data analysis was conducted by NovoExpress software.

**Safety and toxicity assessment**

At the end of treatment, the mice were sacrificed and mouse eye blood was taken to conduct serum biochemistry analysis. Moreover, the heart, liver, spleen, lung, and kidney of the mice were obtained and fixed in 4% paraformaldehyde for further H&E staining and histopathological examination. The indicators for serum biochemistry analysis include total protein (TP), albumin (ALB), alanine aminotransferase (ALT), aspartate aminotransferase (AST), Creatinine (CREA), UREA, uric acid (UA), low-density lipoproteins (LDL), lactate dehydrogenase (LDH), and CK-MB.

**Haematoxylin and eosin staining**

After sacrifice, mouse critical organs mainly including the heart, liver, spleen, lung and kidney were excised and fixed in 4% neutral buffered formalin. The dehydrated tissues were embedded in paraffin. For obtaining 4 µm-thick paraffin tissue sections, the paraffin was sectioned with the microtome. Then, paraffin tissue sections underwent deparaffinization and rehydration. Finally, all the samples were stained with hematoxylin and eosin to further analyze the drug-associated toxicity of JDB 153 on the mice.

**Immunohistochemistry staining**

After sacrifice, all mouse subcutaneous tumor tissues were excised and fixed in 4% neutral buffered formalin and were embedded in paraffin. Subsequently, paraffin-embedded tissues were cut into 4 μm slices and these slices were dewaxed, hydrated and received antigen retrieval. Next, sections were incubated with primary antibodies Ki67 (Abcam, ab16667) and CD31 (Abcam, 56299) overnight at 4 °C. The next day, the staining signal was ascertained with relevant secondary antibodies. Images for IHC analysis were obtained by light microscope.

**References**

1. Fukuoka J, Fujii T, Shih JH, et al. Chromatin remodeling factors and BRM/BRG1 expression as prognostic indicators in non-small cell lung cancer. *Clinical Cancer Research*. 2004;10(13):4314-4324.

2. Yoshizawa A, Fukuoka J, Shimizu S, et al. Overexpression of Phospho-eIF4E Is Associated with Survival through AKT Pathway in Non-Small Cell Lung Cancer. *Clinical Cancer Research*. 2010;16(1):240-248.

3. Ko J, Lung M. In vitro Human Umbilical Vein Endothelial Cells (HUVEC) Tube-formation Assay. *Bio-Protocol*. 2012;2(18)

**Table S1. The clinical characteristics of patients with squamous cell carcinoma.**

| **Variable (%)** | **Squamous cell carcinoma with high p-eIF4E expression (N=35)** | **Squamous cell carcinoma with low p-eIF4E expression**  **(N=26)** | ***P* value** |
| --- | --- | --- | --- |
| **Gender** |  |  | 0.325 |
| Male | 33 (55.9) | 26 (44.1) |  |
| Female | 2 (100.0) | 0 (0.0) |  |
| **Age (median ± SD)** | 63.60 (7.39) | 60.46 (9.91) | 0.282 |
| **Tumor diameter** |  |  | 0.088 |
| <5 cm | 17 (48.6) | 18 (51.4) |  |
| ≥5 cm | 18 (69.2) | 8 (30.8) |  |
| **T stage** |  |  | **0.040** |
| 1 | 0 (0.0) | 2 (100.0) |  |
| 2 | 16 (47.1) | 18 (52.9) |  |
| 3 | 15 (75.0) | 5 (25.0) |  |
| 4 | 4 (80.0) | 1 (20.0) |  |
| **N stage** |  |  | 0.792 |
| 0 | 18 (60.0) | 12 (40.0) |  |
| 1 | 15 (57.7) | 11 (42.3) |  |
| 2 | 2 (40.0) | 3 (60.0) |  |
| **M stage** |  |  | 0.574 |
| 0 | 34 (56.7) | 26 (43.3) |  |
| 1 | 1 (100.0) | 0 (0.0) |  |
| **Clinical stage** |  |  | 0.241 |
| I | 10 (62.5) | 6 (37.5) |  |
| II | 12 (44.4) | 15 (55.6) |  |
| III | 12 (70.6) | 5 (29.4) |  |
| Ⅳ | 1 (100.0) | 0 (0.0) |  |
| **Pathological stage** |  |  | 0.057 |
| I-II | 0 (0.0) | 3 (100.0) |  |
| II | 18 (51.4) | 17 (48.6) |  |
| II-III | 15 (75.0) | 5 (25.0) |  |
| III | 2 (66.7) | 1 (33.3) |  |

**Table S2. The clinical characteristics of patients with adenocarcinoma.**

| **Variable (%)** | **adenocarcinoma with high p-eIF4E expression (N=48)** | **adenocarcinoma with low p-eIF4E expression**  **(N=26)** | ***P* value** |
| --- | --- | --- | --- |
| **Gender** |  |  | 0.084 |
| Male | 24 (55.8) | 19 (44.2) |  |
| Female | 24 (77.4) | 7 (22.6) |  |
| **Age (median ± SD)** | 61.81 (9.22) | 62.54 (11.22) | 0.766 |
| **Tumor diameter** |  |  | 0.425 |
| <5 cm | 36 (67.9) | 17 (32.1) |  |
| ≥5 cm | 12 (57.1) | 9 (42.9) |  |
| **T stage** |  |  | 0.390 |
| 1 | 5 (45.5) | 6 (54.5) |  |
| 2 | 29 (70.7) | 12 (29.3) |  |
| 3 | 10 (58.8) | 7 (41.2) |  |
| 4 | 4 (80.0) | 1 (20.0) |  |
| **N stage** |  |  | 0.673 |
| 0 | 23 (59.0) | 16 (41.0) |  |
| 1 | 10 (66.7) | 5 (33.3) |  |
| 2 | 10 (71.4) | 4 (28.6) |  |
| 3 | 5 (83.3) | 1 (16.7) |  |
| **M stage** |  |  | 1.000 |
| 0 | 47 (64.4) | 26 (35.6) |  |
| 1 | 1 (100.0) | 0 (0.0) |  |
| **Clinical stage** |  |  | 0.293 |
| I | 9 (47.4) | 10 (52.6) |  |
| II | 16 (69.6) | 7 (30.4) |  |
| III | 22 (71.0) | 9 (259.0) |  |
| Ⅳ | 1 (100.0) | 0 (0.0) |  |
| **Pathological stage** |  |  | 0.334 |
| I-II | 4 (100.0) | 0 (0.0) |  |
| II | 26 (60.5) | 17 (39.5) |  |
| II-III | 12 (75.0) | 4 (25.0) |  |
| III | 6 (54.5) | 5 (45.5) |  |


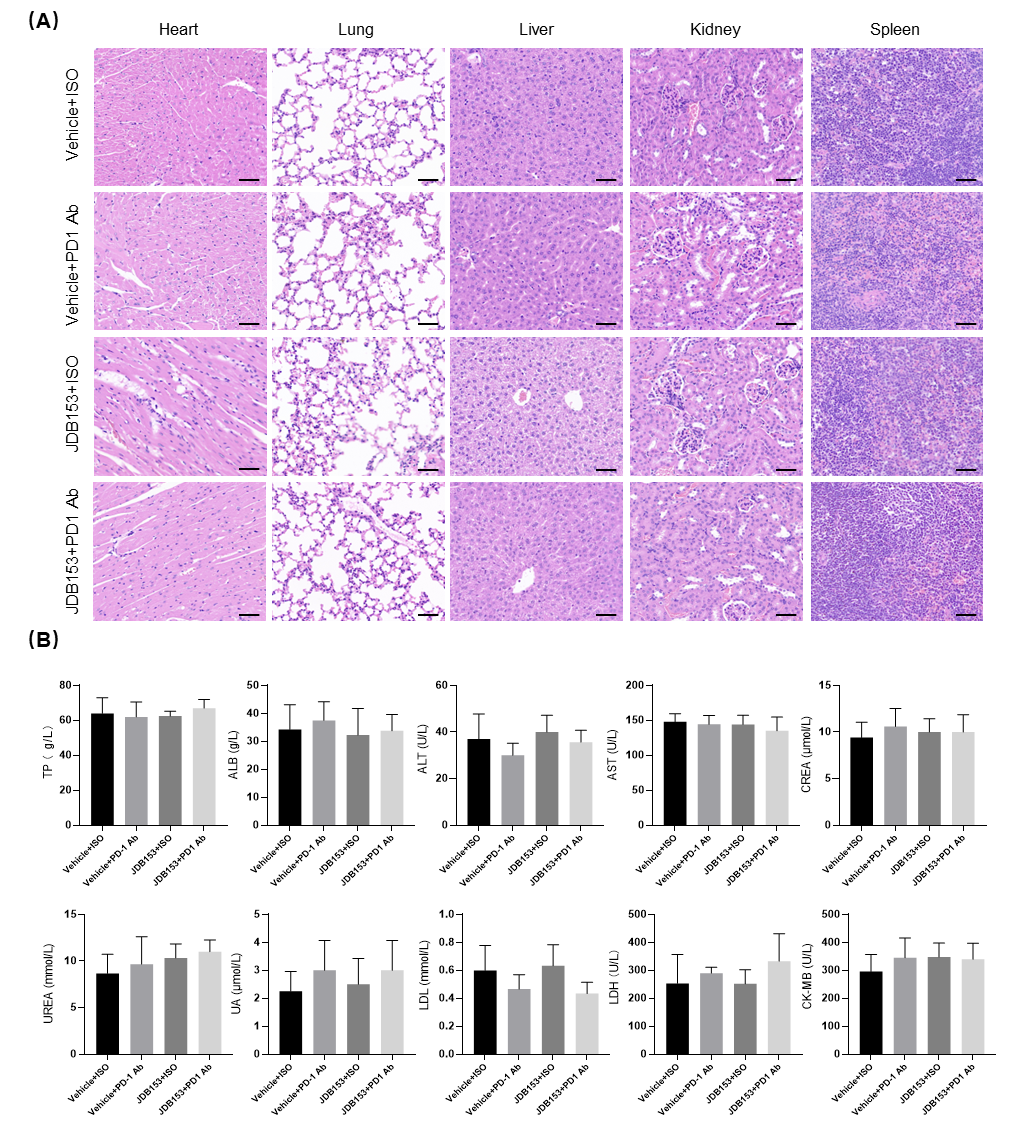


**Figure S1.** The combinational application of JDB153 with PD-1 Ab has shown reliable safety in vivo. (A) Histological examination of major organs (heart, liver, spleen, lung, and kidney) of mice was conducted by H&E staining after treatment to preliminarily evaluate the in vivo potential toxicity of JDB153. (B) The toxicity and safety of the drug were evaluated by biochemical analysis in mice and there were no obvious changes among different groups.


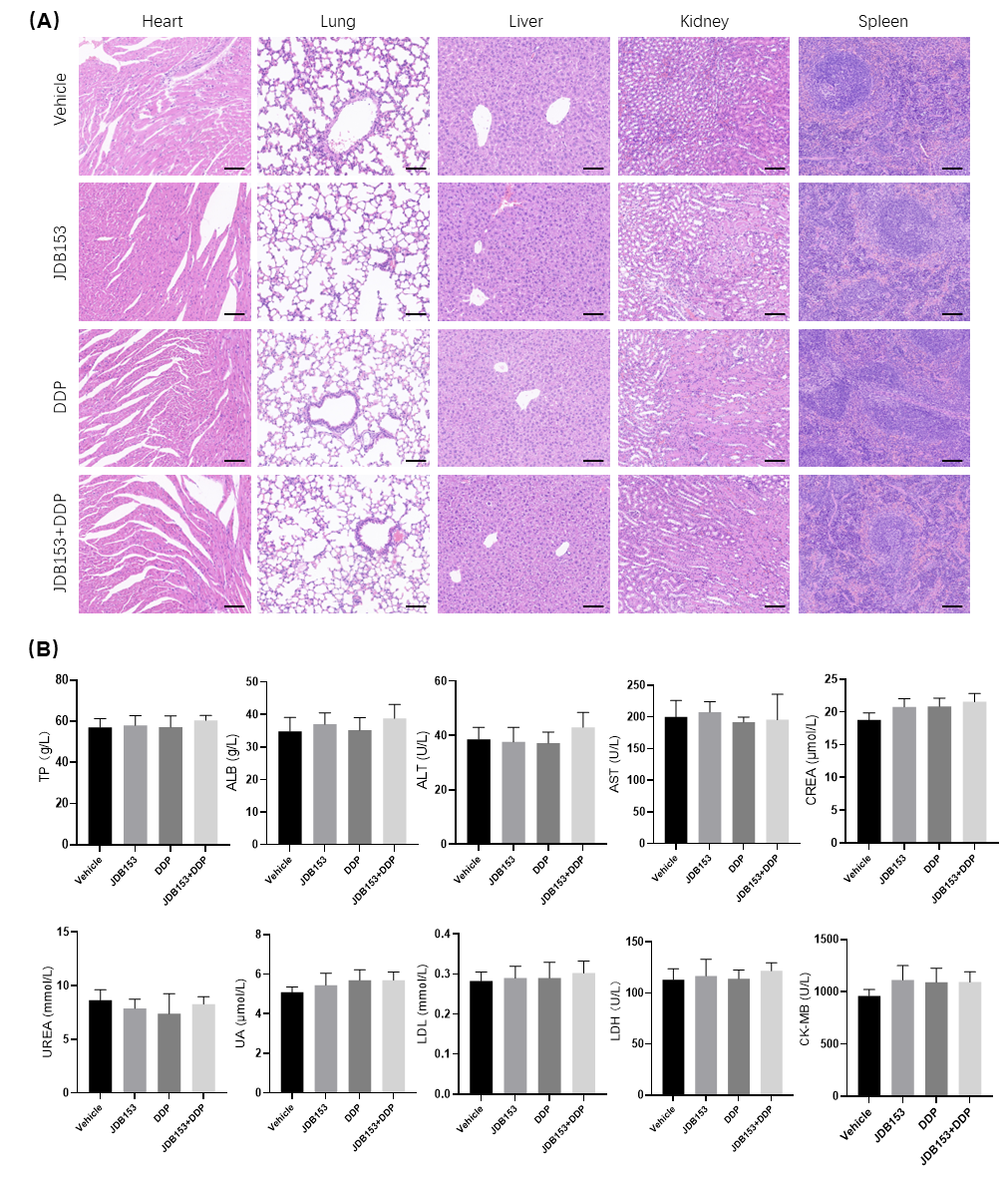


**Figure S2.** The combinational application of JDB153 with DDP has shown reliable safety in vivo. (A) Histological examination of major organs (heart, liver, spleen, lung, and kidney) of mice was conducted by H&E staining after treatment to preliminarily evaluate the in vivo potential toxicity of JDB153. (B) The toxicity and safety of the drug were evaluated by biochemical analysis in mice and there were no obvious changes among different groups.
